# Self-Assembled Thin-Layer Glycomaterials With a Proper Shell Thickness for Targeted and Activatable Cell Imaging

**DOI:** 10.3389/fchem.2019.00294

**Published:** 2019-05-08

**Authors:** Chao Zhang, Guanzhen Wang, Hai-Hao Han, Xi-Le Hu, Robert A. Field, Guo-Rong Chen, Jia Li, Bing Ye, Xiao-Peng He, Yi Zang

**Affiliations:** ^1^Emergency Department, Jinan Children's Hospital, Jinan, China; ^2^State Key Laboratory of Drug Research, National Center for Drug Screening, Chinese Academy of Sciences, Shanghai Institute of Materia Medica, Shanghai, China; ^3^University of Chinese Academy of Sciences, Beijing, China; ^4^Key Laboratory for Advanced Materials and Feringa Nobel Prize Scientist Joint Research Center, School of Chemistry and Molecular Engineering, East China University of Science and Technology, Shanghai, China; ^5^Department of Biological Chemistry, John Innes Centre, Norwich Research Park, Norwich, United Kingdom

**Keywords:** fluorescence, precision, imaging, activatable, receptor

## Abstract

The construction of targeted and activatable materials can largely improve the precision of disease diagnosis and therapy. However, the currently developed systems either target a transmembrane antigen or are activatable to release imaging and/or therapeutic reagents intracellularly. Here, we develop a simple thin-layer glycomaterial through the self-assembly between fluorescent glycoprobes, in which the carbohydrate-targeting reagent and the fluorophore are linked to each other by polyethylene glycol with a suitable chain length, and thin-layer manganese dioxide. The fluorogenic material developed is both capable of targeting a transmembrane glycoprotein receptor and fluorescently activatable by intracellular biothiols. The shell thickness of the material was determined to be important for achieving the biothiol-induced activation of fluorescence. This research might provide insight into the development of precision-enhanced self-assembled materials for disease theranostics.

## Introduction

Carbohydrate–protein interactions are responsible for the activation of many biological and disease-relevant signaling pathways (Lee and Lee, 1995). During the process of a certain number of diseases, transmembrane receptors that are selective for carbohydrates (monosaccharides or oligosaccharides) are overexpressed (Kampen, 2011). As a result, glycomaterials, which are prepared by covalently or non-covalently conjugating carbohydrates to a variety of different material substrates including polymers, nanoparticles, and thin-layer materials, have been developed for targeted disease diagnosis and therapy (Ji et al., 2016; Zhang et al., 2017; Fu et al., 2018).

Recently, the use of thin-layer materials, such as graphene oxide and graphene-like materials, for biomedical applications has emerged as a topical research area (Chung et al., 2013; Shareena et al., 2018). Among the advanced materials developed, thin-layer molybdenum disulfides and oxides have been proven to be of good biocompatibility with low *in vitro* and *in vivo* toxicity to construct theranostic materials (Liu et al., 2014, 2015; He and Tian, 2016; Yadav et al., 2019). Thin-layer manganese dioxide (MnO_2_), which can be readily degraded to form manganese ions, has been extensively used to construct activatable sensing and therapeutic materials in response to the reducing microenvironments or low pH inside cancer cells (Zhao et al., 2014; Fan et al., 2015; Chen et al., 2016).

While previous studies mainly focused on the development of materials that can target a transmembrane antigen or are activatable for controlled release of imaging and therapeutic agents, here we develop a thin-layer glycomaterial for both targeted and activatable imaging of cells. Self-assembly between fluorescent glycoprobes and thin-layer MnO_2_ produces fluorogenic glycomaterials, which can target a transmembrane glycoprotein receptor to deliver the glycoprobes inside cells. Then, degradation of the thin-layer MnO_2_ backbone by intracellular biothiols activates the glycoprobe fluorescence, enabling the targeted, activatable functional cell imaging. Importantly, we demonstrate that the shell thickness is crucial for achieving the biothiol-responsive fluorescence activation of the thin-layer glycomaterials.

## Results and Discussion

**Figure 1:**
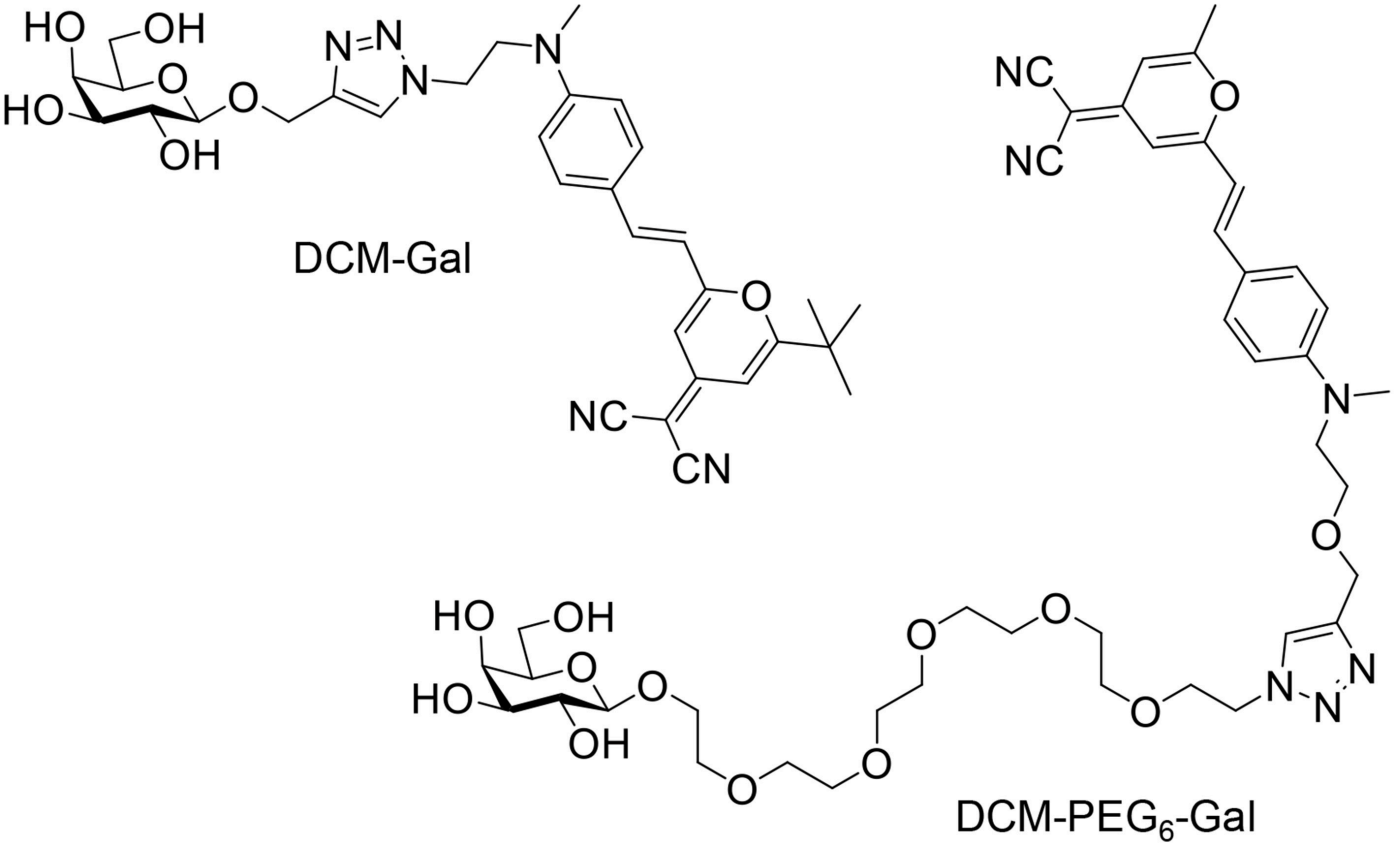
Structure of the glycoprobes used for self-assembly with thin-layer MnO_2_.

**Figure 2:**
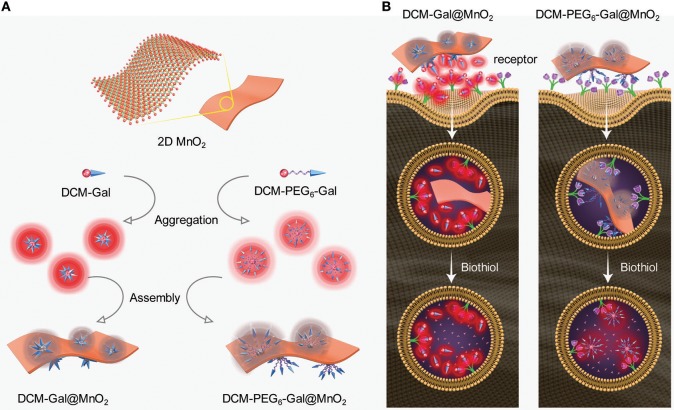
Schematic illustration of **(A)** aggregation and then self-assembly of the glycoprobes with thin-layer MnO_2_, producing the thin-layer glycomaterials with different shell thicknesses, and **(B)** the different fluorescence activation mode of the glycomaterials after endocytosis by cells that express asialoglycoprotein receptors.

Two DCM (dicyanomethylene-4H-pyran)-based glycoprobes [DCM-Gal (Ji et al., 2016) and DCM-PEG_6_-Gal] with linkers of different lengths connecting a DCM and a galactose epitope were used (Figure 1). An experimental section and original NMR spectral copies of new compounds are presented in Supplementary Material. The presence of a hexa-PEG linkage in the structure of DCM-PEG_6_-Gal could facilitate the formation of a PEG shell on the surface of thin-layer materials in order to enhance the stability of the material in complex biological environments (Figure 2A). We envision that while the material composite formed between DCM-Gal and thin-layer MnO_2_ might dissociate directly after interaction with the asialoglycoprotein receptor (ASGPr) that selectively recognizes galactoconjugates, that formed between DCM-PEG_6_-Gal and thin-layer MnO_2_ could be more stable during receptor-mediated endocytosis for stimuli-activated fluorescence imaging (Figure 2B).

**Figure 3:**
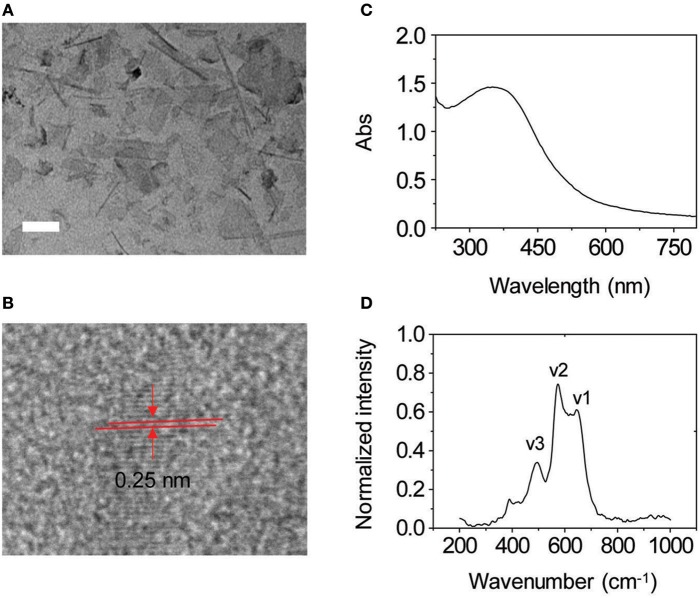
**(A)** Representative high-resolution transmission electron microscopic (HRTEM) image of thin-layer MnO_2_ (scale bar = 50 nm). **(B)** An enlarged view of an HRTEM image of thin-layer MnO_2_. **(C)** UV–vis absorption spectrum of thin-layer MnO_2_ (100 μg ml^−1^ dissolved in Tris-HCl buffer). **(D)** Raman spectrum of thin-layer MnO_2_ (100 μg ml^−1^ dissolved in Tris-HCl buffer).

**Figure 4:**
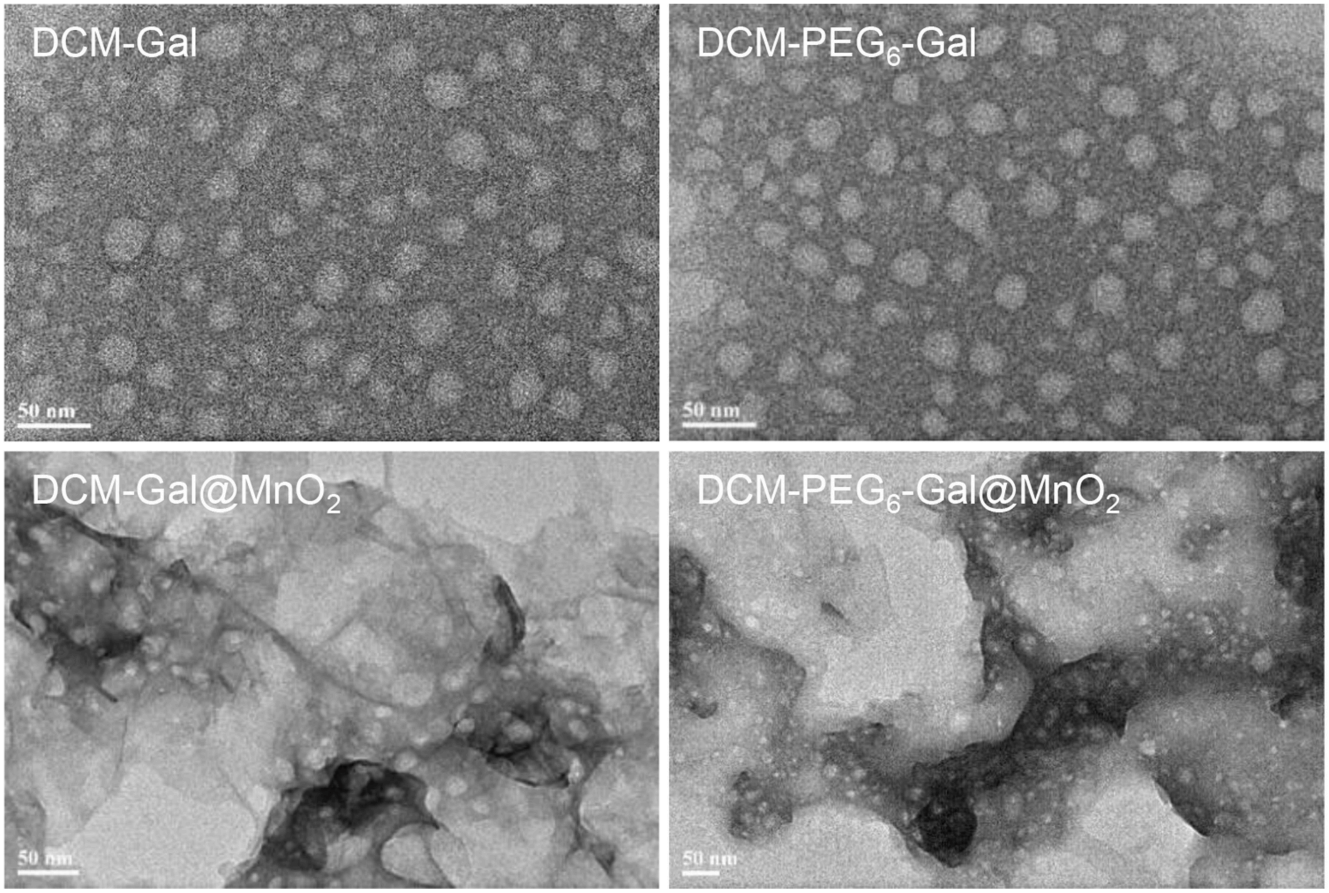
Representative HRTEM images of DCM-Gal, DCM-PEG_6_-Gal, DCM-Gal@MnO_2_ (DCM-Gal/MnO_2_ = 10 μM/10 μg ml^−1^ dissolved in Tris-HCl buffer), and DCM-PEG_6_-Gal@MnO_2_ (DCM-PEG_6_-Gal/MnO_2_ = 10 μM/10 μg ml^−1^ dissolved in Tris-HCl buffer).

To prove our hypothesis, the glycoprobes were used for self-assembly in Tris-HCl buffer with thin-layer MnO_2_ prepared by the previously reported method (Zhao et al., 2014). In its representative high-resolution transmission electron microscopy (HRTEM) images, we observed thin-flake objects, suggestive of the formation of thin-layer MnO_2_ (Figure 3A). The orthogonal distance (~0.25 nm) between two consecutive slabs of [MnO_6_] is characteristic of the typical birnessite-type MnO_2_ (Figure 3B; Kim et al., 2017). In the UV spectrum of the thin-layer MnO_2_, a predominant absorbance peak at ca. 380 nm was detected (Figure 3C), which is attributable to the d–d transition of Mn ions in the MnO_6_ octahedra of the thin-layer material (Kai et al., 2008). Raman spectroscopy was also used for material characterization. Three typical bands at 647, 575, and 497 cm^−1^ were observed, which are characteristic of the ν_1_ (the symmetric stretching vibration of the Mn–O bond in the MnO_6_ octahedral plane), ν_2_ (the stretching vibration mode of Mn–O in the MnO_6_ octahedral basal plane), and ν_3_ (the deformation mode of the metal–oxygen chain of Mn–O–Mn in the MnO_2_ octahedral lattice) vibrational features of thin-layer MnO_2_, respectively (Figure 3D; Julien et al., 2003, 2004). We also observed that both DCM-Gal and DCM-PEG_6_-Gal form nanoparticles (Figure 4), whereas after assembly, the particles were determined to be adhered onto the surface of thin-layer MnO_2_ (Figure 4).

**Figure 5:**
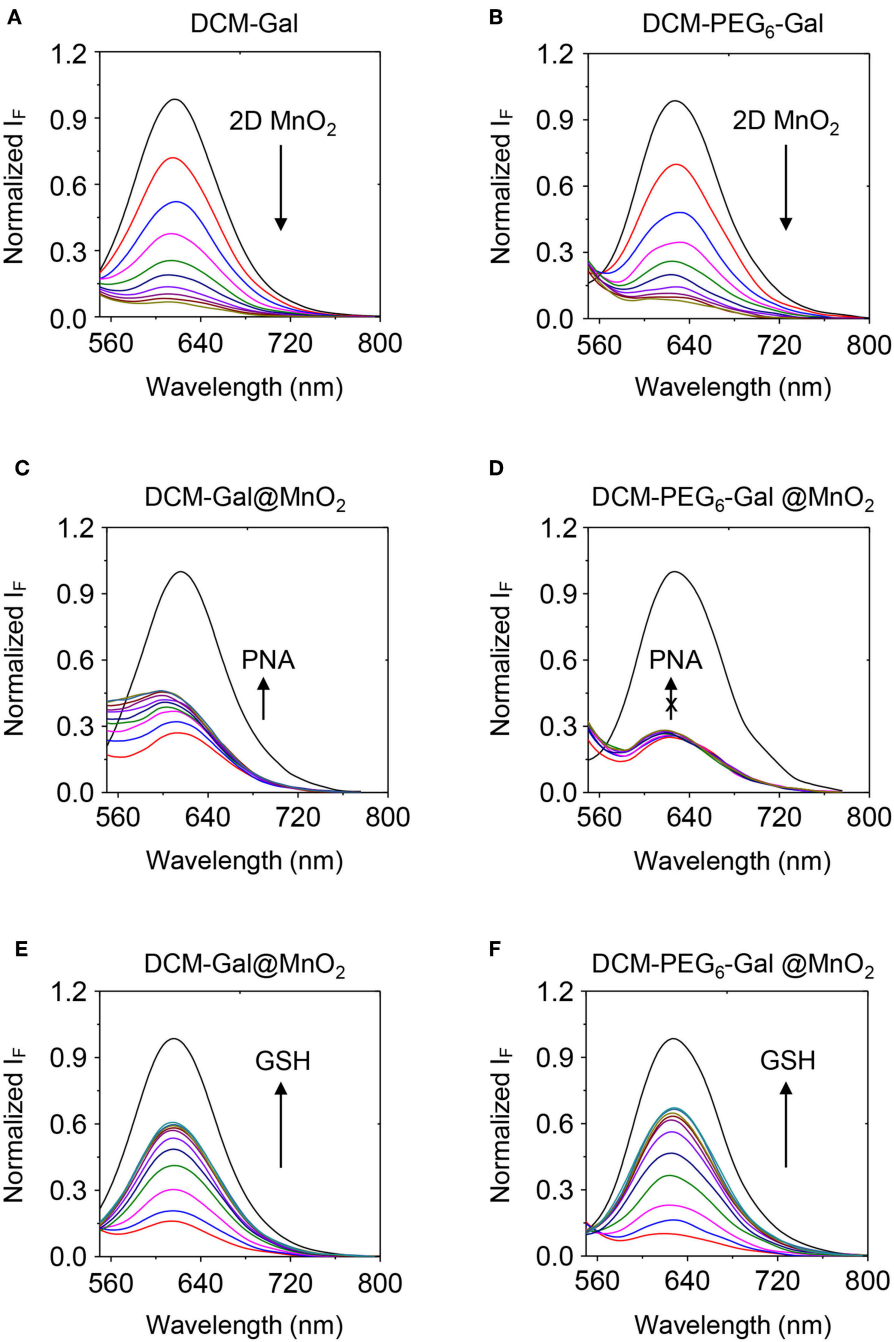
Fluorescence spectra of **(A)** DCM-Gal (10 μM) and **(B)** DCM-PEG_6_-Gal (10 μM) with increasing thin-layer MnO_2_ (from top to bottom curve: 0–40 μg ml^−1^, interval: 4 μg ml^−1^). Fluorescence spectra of **(C)** DCM-Gal@MnO_2_ (DCM-Gal/MnO_2_ = 10 μM/40 μg ml^−1^) and **(D)** DCM-PEG_6_-Gal@MnO_2_ (DCM-PEG_6_-Gal/MnO_2_ = 10 μM/20 μg ml^−1^) with increasing PNA (peanut agglutinin, from bottom to top curve: 0–40 μM, interval: 5 μM). Fluorescence spectra of **(E)** DCM-Gal@MnO_2_ (DCM-Gal/MnO_2_ = 10 μM/40 μg ml^−1^) and **(F)** DCM-PEG_6_-Gal@MnO_2_ (DCM-PEG_6_-Gal/MnO_2_ = 10 μM/40 μg ml^−1^) with increasing GSH (γ-glutathione, from bottom to top curve: 0–500 μM, interval: 50 μM).

Subsequently, fluorescence spectroscopy was used for the analysis of the self-assembly. We observed a gradually decreased fluorescence of both DCM-Gal (Figure 5A) and DCM-PEG_6_-Gal (Figure 5B) in the presence of increasing thin-layer MnO_2_. This suggests the adsorption of the glycoprobes onto the surface of the material, leading to fluorescence quenching (Zhao et al., 2014). To test its stability toward a carbohydrate-binding protein, we added peanut agglutinin (PNA) that selectively recognizes the galactose epitopes on the surface of the material composite. Interesting, while a gradual fluorescence enhancement was observed for the DCM-Gal@MnO_2_ group with increasing PNA (Figure 5C), which is in accordance with our previous observations that complexation between glycoprobe and PNA competitively removes the probe molecules from the surface of the quenching material, the quenched fluorescence of DCM-PEG_6_-Gal remained almost unchanged (Figure 5D). This suggests the importance of the hexa-PEG shell for the protection of the material composite from disassociation upon interaction with a galactose-selective lectin. In contrast, the fact that the presence of GSH led to the fluorescence enhancement of both glycomaterials, which is the result of degradation of the thin-layer MnO_2_ backbone, suggests their ability for activatable fluorescence sensing and imaging (Figures 5E,**F**).

**Figure 6:**
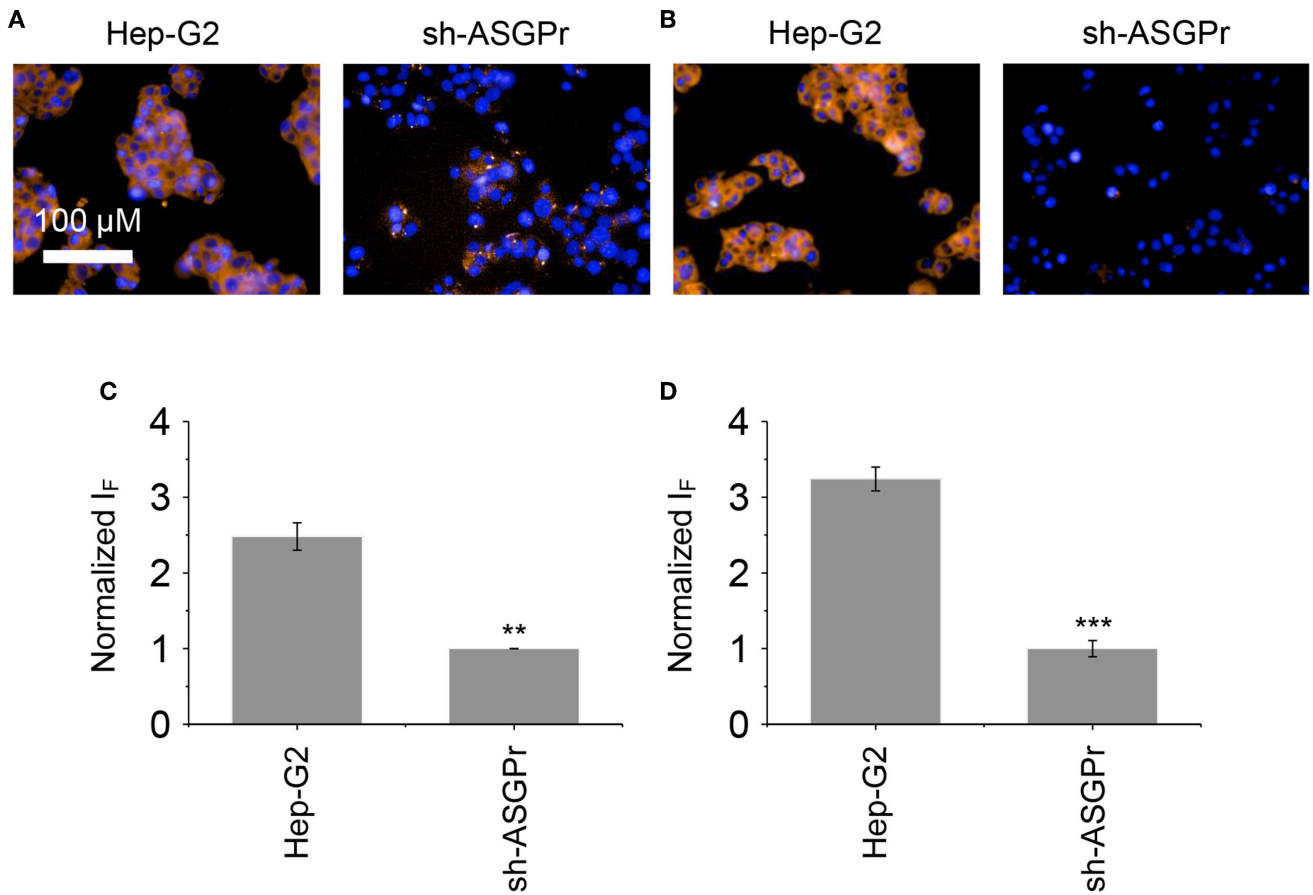
Fluorescence imaging **(A)** and quantification **(C)** of Hep-G2 and sh-ASGPr cells after incubation with DCM-Gal@MnO_2_ (DCM-Gal/MnO_2_ = 10 μM/32 μg ml^−1^). Fluorescence imaging **(B)** and quantification **(D)** of Hep-G2 and sh-ASGPr cells after incubation with DCM-PEG_6_-Gal@MnO_2_ (DCM-PEG_6_-Gal/MnO_2_ = 10 μM/32 μg ml^−1^). **P < 0.01, ***P < 0.005. Error bars mean S. D. (*n* = 3). Excitation and emission channels used were 460–490 and 560–630 nm, respectively. Cell nuclei were stained by Hoechst 33342 (excitation and emission channels were 360–400 and 410–480 nm, respectively).

**Figure 7:**
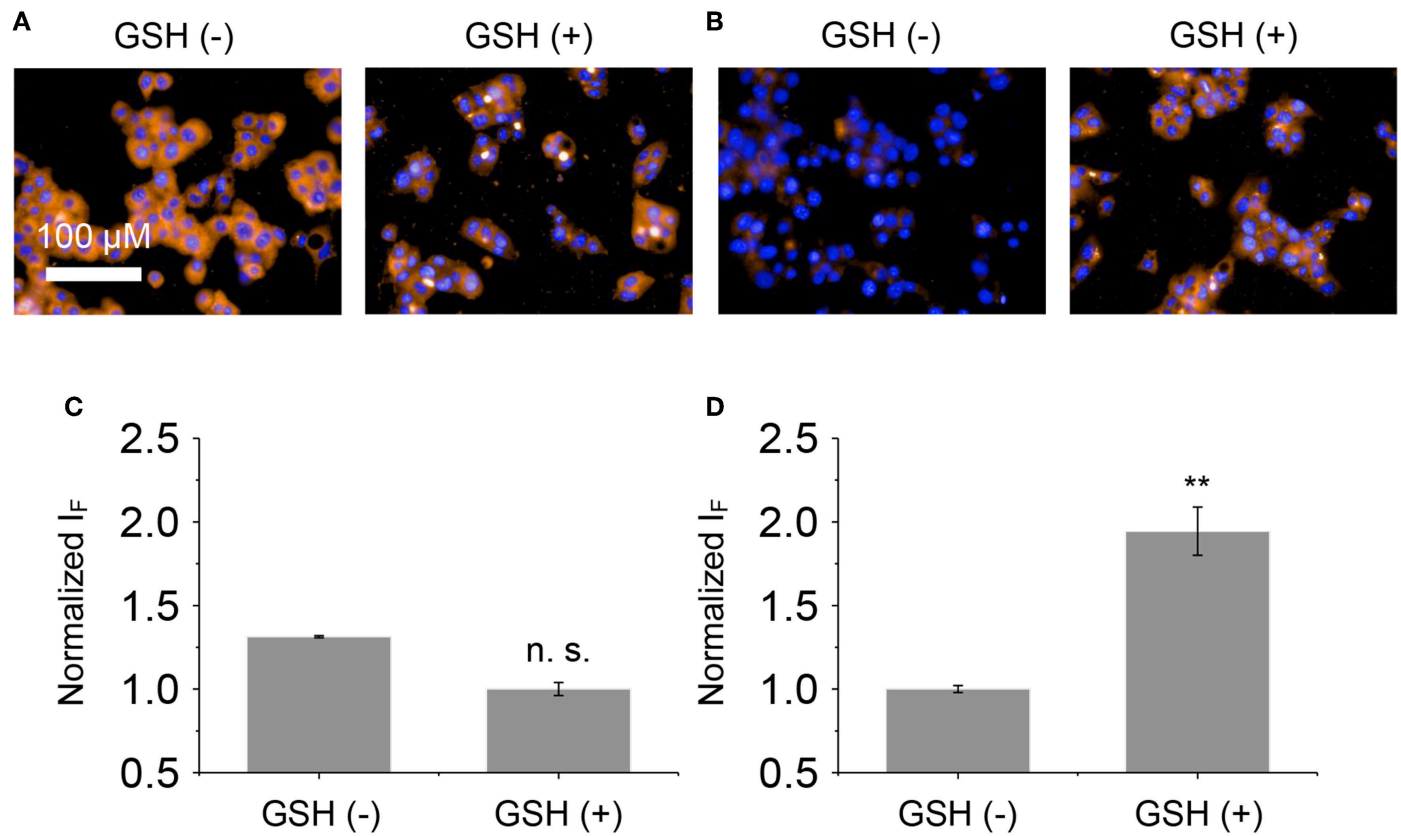
Fluorescence imaging **(A)** and quantification **(C)** of Hep-G2 treated with DCM-Gal@MnO_2_ (DCM-Gal/MnO_2_ = 10 μM/32 μg ml^−1^) pretreated with NEM (N-ethylmaleimide is a known GSH quencher, 500 μM) or GSH (300 μM). Fluorescence imaging **(B)** and quantification **(D)** of Hep-G2 treated with DCM-PEG_6_-Gal@MnO_2_ (DCM-PEG_6_-Gal/MnO_2_ = 10 μM/32 μg ml^−1^) pretreated with NEM (500 μM) or GSH (300 μM). n.s., not significant; **P < 0.01. Error bars mean S. D. (*n* = 3). Excitation and emission channels used were 460–490 and 560–630 nm, respectively. Cell nuclei were stained by Hoechst 33342 (excitation and emission channels were 360–400 and 410–480 nm, respectively).

Next, the glycomaterials were used for cell imaging. Hep-G2 cells that highly express ASGPr as well as GSH, and a previously established Hep-G2 cell line with a reduced ASGPr expression by gene transfection (Fu et al., 2018), were used to test the receptor-targeting capacity of the materials. We determined that the fluorescence of both materials was produced mainly in Hep-G2 rather than in sh-ASGPr cells, suggesting their good receptor-targeting property because of the exposure of galactose epitopes on the surface (Figure 6; Burgess et al., 1992). Then, we used Hep-G2 cells with a depleted GSH concentration by pretreatment with NEM (a known GSH scavenger) to measure the fluorescence activity of the materials. A similar level of fluorescence was determined in Hep-G2 cells with or without GSH for the DCM-Gal@MnO_2_ group (Figures 7A,**C**). In contrast, the fluorescence of DCM-PEG_6_-Gal@MnO_2_ in Hep-G2 cells with endogenous GSH was much stronger than in those with depleted GSH (Figures 7B,**D**). These results preliminarily suggest that while the DCM-Gal@MnO_2_ ensemble disassociates upon interaction with ASGPr, DCM-PEG_6_-Gal@MnO_2_, because of the presence of a hexa-PEG shell, remained much more stable upon receptor-mediated endocytosis. However, the subsequent presence of a high concentration of intracellular GSH led to material degradation, thus enabling activatable fluorescence imaging (Figure 2B).

## Conclusions

We have shown in this research that by properly modulating the shell thickness of self-assembled, thin-layer glycomaterials can enable targeted and activatable imaging of cells. The glycomaterial coated with a hexa-PEG shell can effectively protect the material ensemble from disassociation after incubation with a lectin that selectively recognizes the carbohydrate epitopes on the material surface. In the subsequent cell imaging assay, we also observed that the fluorescence activation of the thickly shelled glycomaterial was dependent on the presence intracellular biothiols, while that which lacks the protective shell was directly dependent on the expression of transmembrane glycoprotein receptors irrespective of the intracellular GSH concentration. This implies the importance of properly adjusting the shell thickness of self-assembled thin-layer materials in order to enhance the precision of functional cell imaging. We are currently using this concept for the construction of other thin-layer MnO_2_-based materials for the analysis of the biothiol level in different types of cancer cells such as leukemia cells.

## Data Availability

The raw data supporting the conclusions of this manuscript will be made available by the authors, without undue reservation, to any qualified researcher.

## Author Contributions

All authors listed have made a substantial, direct and intellectual contribution to the work, and approved it for publication.

### Conflict of Interest Statement

The authors declare that the research was conducted in the absence of any commercial or financial relationships that could be construed as a potential conflict of interest.
