# Supplementary material for: Self-Assembled Thin-Layer Glycomaterials With a Proper Shell Thickness for Targeted and Activatable Cell Imaging
Source: Front Chem. 2019 May 8;7:294. doi: 10.3389/fchem.2019.00294 (PMC6517789; doi:10.3389/fchem.2019.00294)
Supplement: Supplementary file 1 [file Data_Sheet_1.docx]

**Supporting Information**

**Self-assembled thin-layer glycomaterials with an appropriate shell thickness for targeted and activatable cell imaging**

Chao Zhang,^1,a^ Guanzhen Wang ^3,5,a^ Hai-Hao Han,^2,a^ Xi-Le Hu, Robert A. Field,^4^ Guo-Rong Chen,^2^ Jia Li,^3^ Bing Ye,^1,^* Xiao-Peng He,^2^* & Yi Zang^3^*

^1^ Jinan Children’s Hospital, Emergency Department, Jinan 250022, Shandong, PR China

^2^ Key Laboratory for Advanced Materials and Feringa Nobel Prize Scientist Joint Research Center, School of Chemistry and Molecular Engineering, East China University of Science and Technology, 130 Meilong Road, Shanghai 200237, PR China

^3^ National Center for Drug Screening, State Key Laboratory of Drug Research, Shanghai Institute of Materia Medica, Chinese Academy of Sciences, 189 Guo Shoujing Rd., Shanghai 201203, PR China

^4^ Department of Biological Chemistry, John Innes Centre, Norwich Research Park, Norwich NR4 7UH, UK

^5^ University of Chinese Academy of Science, No.19A YuquanRoad, Beijing100049, P.R.China

^a^ Equal contribution

Email: [yzang@simm.ac.cn](mailto:yzang@simm.ac.cn) (Y. Zang), [xphe@ecust.edu.cn](mailto:xphe@ecust.edu.cn) (X.-P. He), [13583184890@163.com](mailto:13583184890@163.com) (B. Ye)

**Contents list**

S1. Experimental section

S2. Original spectral copies of new compounds

S3. Additional references

**S1. Experimental section**

**General.** All purchased chemicals and reagents are of analytical grade. γ-Glutathione (GSH) and peanut agglutinin (PNA) were purchased from Sigma-Aldrich. ^1^H NMR and ^13^C NMR spectra were recorded on a Bruker AM 400 MHz spectrometer with tetramethylsilane (TMS) as internal reference. UV-vis Absorption spectra were measured on a Varian Cary 500 UV-Vis spectraphotometer. High resolution mass spectra (HRMS) were recorded with a Waters Micromass LCT mass spectrometer. Thin-layer MnO_2_ was prepared according to a previous literature report (Zhao et al., 2014).

**Self-assembly of thin-layer glycomaterial.** To an aqueous suspension of thin-layer MnO_2_ (1 mL, 1 mg mL^-1^) glycoprobe **g** (100 μL, 10 mM) was added. The resulting mixture was sonicated for 20 min. Then the mixture was centrifuged at 10000 rpm for 20 min to remove excessive compounds. The residue was re-dissolved in Tris-HCl buffer and used as-is.

**HRTEM.** A droplet of 2D MnO_2_ (10 μg mL^-1^; Tris-HCl buffer), 2D glycocluster (glycoprobe/MnO_2_ = 10 µM/10 μg mL^-1^) or glycoprobe (10 µM) was dropped onto 200 mesh holey carbon copper grids. Then, the images were recorded with JEOL 2100 equipped with a Gatan Orius charged-coupled device camera and Tridiem energy filter operating at 200 kV.

**Raman spectroscopy.** Raman spectra of thin-layer MnO_2_ (100 μg mL^-1^, Tris-HCl buffer) was obtained using a Renishaw In Via Reflex Raman system (Renishaw plc, Wotton-under-Edge, UK) employing a grating spectrometer with a Peltier-cooled charge-coupled device detector coupled to a confocal microscope. The raw data obtained were processed with Renishaw WiRE 3.2 software. The Raman scattering was excited by an argon ion laser (I = 514.5 nm).

**Cell culture**. Hep-G2 cells were maintained in a Dulbecco’s Modified Eagle’s Medium (Invitrogen, Carlsbad, CA, USA) supplemented with 10% fetal bovine serum (Gibco, Gland Island, NY, USA) in a humidified atmosphere of 5% CO_2_ and 95% air at 37 ̊C and split when the cells reached 90% confluency.

**Fluorescence imaging of cells.** Cells were cultured in growth medium supplemented with 10% FBS. Then, cells were seeded on a black 96-well microplate with optically clear bottom (Greiner bio-one, Germany) overnight, and then incubated with DCM-Gal@MnO_2_ or DCM-PEG_6_-Gal@MnO_2_ for 15 min. The cells nuclei were stained with Hoechst 33342 (5 μg mL^-1^) at 37 °C in a humidified atmosphere of 5% CO_2_ in air for 5 min. Then, cells were washed with PBS three times. The fluorescence images were recorded using an Operetta high-content imaging system (Perkinelmer, US), and was quantified and plotted by Columbus analysis system (Perkinelmer, US).

**Synthesis of glycoprobe.**

**Scheme S1.** Reagents and conditions: (I) NaH/DMF; (II) Piperidine/PrOH, (1:5, v/v) at 60 °C.

**Synthesis of c (a (Yan et al., 2017) was prepared according to a previous protocol).** To a solution of **a** (300 mg, 1.7 mmol) in dry DMF (20 mL) were added NaH (107.2 mg, 3.3 mmol) and **b** (262.2 uL, 3.3 mmol). The mixture was stirred for 1 h and then diluted with CH_2_Cl_2_ and washed with brine. The organic layer was dried over MgSO_4_, filtered and concentrated in vacuum to give a crude product, which was then purified by column chromatography (PE (petroleum ether)/EA (EtOAc) = 3:1, v/v) to obtain **c** as a yellow syrup (281.5 mg, 68%). *R*_f_ 0.70 (PE/EA = 2:1, v/v).

^1^H NMR (400 MHz, chloroform-*d*_6_) *δ* 9.74 (s, 1H), 7.74 (d, *J* = 8.8 Hz, 2H), 6.75 (d, *J* = 8.8 Hz, 2H), 4.16 (s, 2H), 3.73 (t, *J* = 6.0, 5.2 Hz, 2H), 3.68 (t, *J* = 5.2, 6.0 Hz, 2H), 3.11 (s, 3H), 2.43 (t, *J* = 2.4, 2.4 Hz, 1H); ^13^C NMR (101 MHz, chloroform-*d*_6_) *δ* 190.3, 153.5, 132.1, 125.3, 111.1, 79.4, 74.8, 67.2, 58.5, 51.9, 39.3. HRMS (ESI, *m/z*): [M+Na]^+^ calcd for C_15_H_25_N_2_O_2_Na^+^ 240.1000, found 240.0988.

**Synthesis of e.** To a solution of **c** (150 mg, 0.6 mmol) in piperidine (3 mL) and PrOH (15 mL) was added **d** (133.6 mg, 0.6 mmol). The mixture was stirred over night at 60 °C, and then concentrated in vacuum to give a crude product, which was then purified by column chromatography (PE/EA = 4:1, v/v) to obtain **e** as a yellow syrup (176.3 mg, 76%). *R*_f_ 0.30 (PE/EA = 3:1, v/v).

^1^H NMR (400 MHz, chloroform-*d*_6_) *δ* 7.41 (d, *J* = 8.7 Hz, 2H), 7.35 (d, *J* = 15.8 Hz, 1H), 6.74 (d, *J* = 7.9 Hz, 2H), 6.59 (d, *J* = 1.9 Hz, 1H), 6.48-6.44 (m, 2H), 4.16 (d, *J* = 2.4 Hz, 2H), 3.73 (t, *J* = 5.7 Hz, 2H), 3.64 (t, *J* = 5.7 Hz, 2H), 3.08 (s, 3H), 2.43 (t, *J* = 2.4 Hz, 1H), 2.38 (s, 3H); ^13^C NMR (101 MHz, chloroform-*d*_6_) *δ* 161.7, 160.5, 156.5, 150.7, 138.6, 129.7, 122.5, 115.6, 112.6, 112.0, 106.2, 105.5, 79.4, 74.7, 67.3, 58.6, 57.4, 52.0, 39.1, 29.7, 19.9. HRMS (ESI, *m/z*): [M+H]^+^ calcd for C_23_H_22_N_3_O_2_^+^ 372.1712, found 372.1707.

**Scheme S2.** Reagents and conditions: (I) CuSO_4_·5H_2_O, sodium ascorbate in CH_2_Cl_2_/H_2_O/t-BuOH (2:1:1, v/v) at 60 °C.

**Synthesis of g by Cu(I)-catalyzed azide-alkyne 1,3-dipolar cycloaddition (Scheme S2).** To a soln. of azido glycoside **f** (prepared according to a previous report) (Hu et al., 2016) (500 mg, 1.06 mmol) and alkyne **e** (395.5 mg, 1.06 mmol) in a solvent mixture of CH_2_Cl_2_/H_2_O/t-BuOH (2:1:1, v/v) were added CuSO_4_·5H_2_O and Na ascorbate. The mixture was stirred at 60 °C for 12 h under nitrogen. The resulting mixture was diluted with CH_2_Cl_2_ and washed with brine. The combined organic layer was dried over MgSO_4_, filtered, and concentrated in vacuum to give a crude product, which was purified by column chromatography (CH_2_Cl_2_/MeOH = 10:1, v/v) to afford **g** as a yellow solid (756.8 mg, 85%). *R*_f_ 0.30 (CH_2_Cl_2_/MeOH = 10:1, v/v).

^1^H NMR (400 MHz, CD_3_OD) *δ* 7.91 (s, 1H), 7.46-7.37 (m, 3H), 6.73-6.70 (m, 3H), 6.58-6.57 (d, *J* = 7.6 Hz, 1H), 6.48-6.47 (d, *J* = 5.2 Hz, 1H), 4.59 (s, 2H), 4.54-4.51 (m, 2H), 4.24-4.22 (d, *J* = 7.6 Hz, 1H), 4.01-3.96 (m, 1H), 3.85-3.82 (m, 3H), 3.78-3.68 (m, 5H), 3.66-3.63 (m, 4H), 3.62-3.59 (m, 9H), 3.58-3.53 (m, 7H), 3.52-3.43 (m, 3H), 3.03 (s, 3H), 2.39 (s, 3H); ^13^C NMR (101 MHz, CD_3_OD) *δ* 164.6, 162.8, 158.4, 152.4, 145.8, 140.3, 131.1, 125.9(2), 123.9, 116.8, 113.6, 113.1, 106.8, 106.2, 105.1, 76.7, 74.9, 72.5, 71.6, 71.5(3), 71.4, 70.3(2), 69.6, 68.9, 65.1, 62.6, 56.3, 52.9, 51.4, 39.5, 19.9. HRMS (ESI, m/z): [M+H]^+^ calcd for C_41_H_57_N_6_O_13_^+^ 841.3984, found 841.3988.

**S2. Original spectral copy of new compounds**


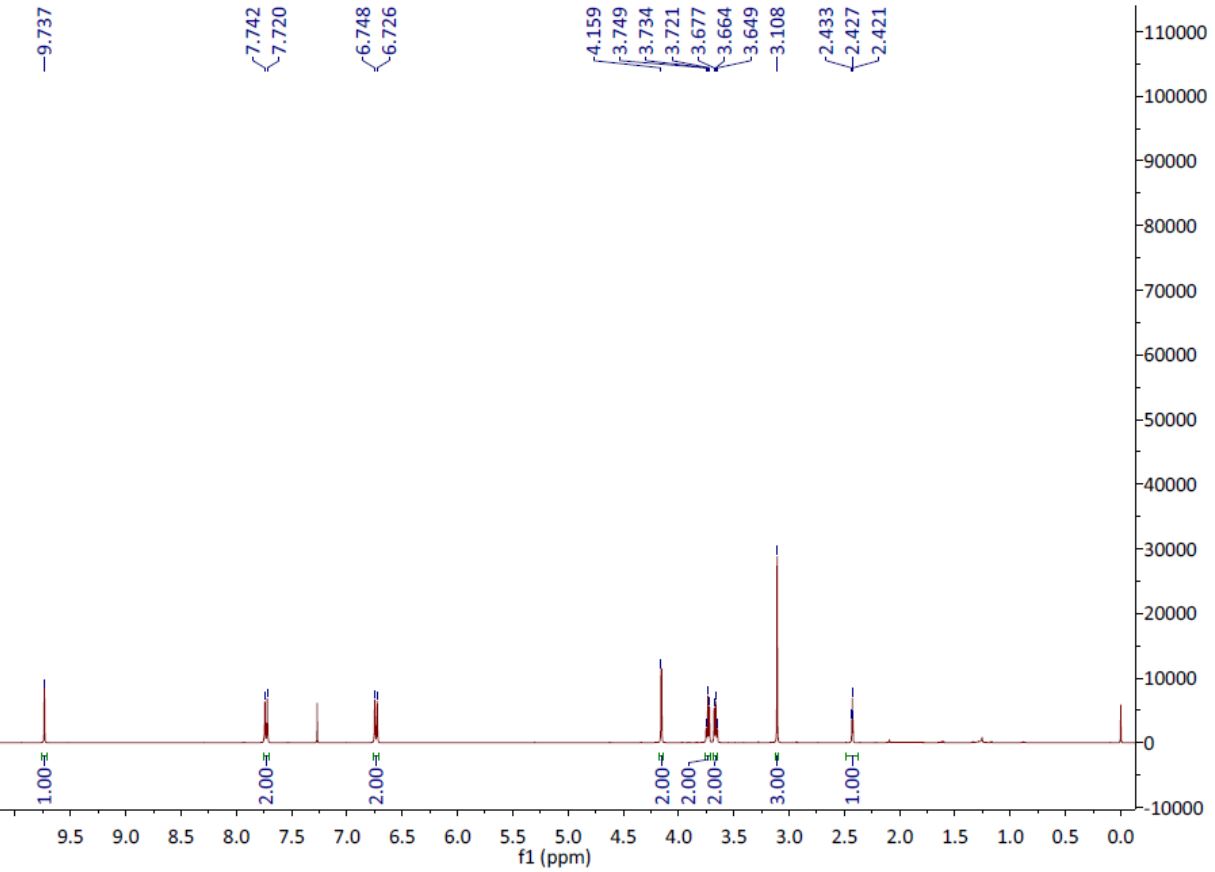


^1^H NMR of **c**.

**
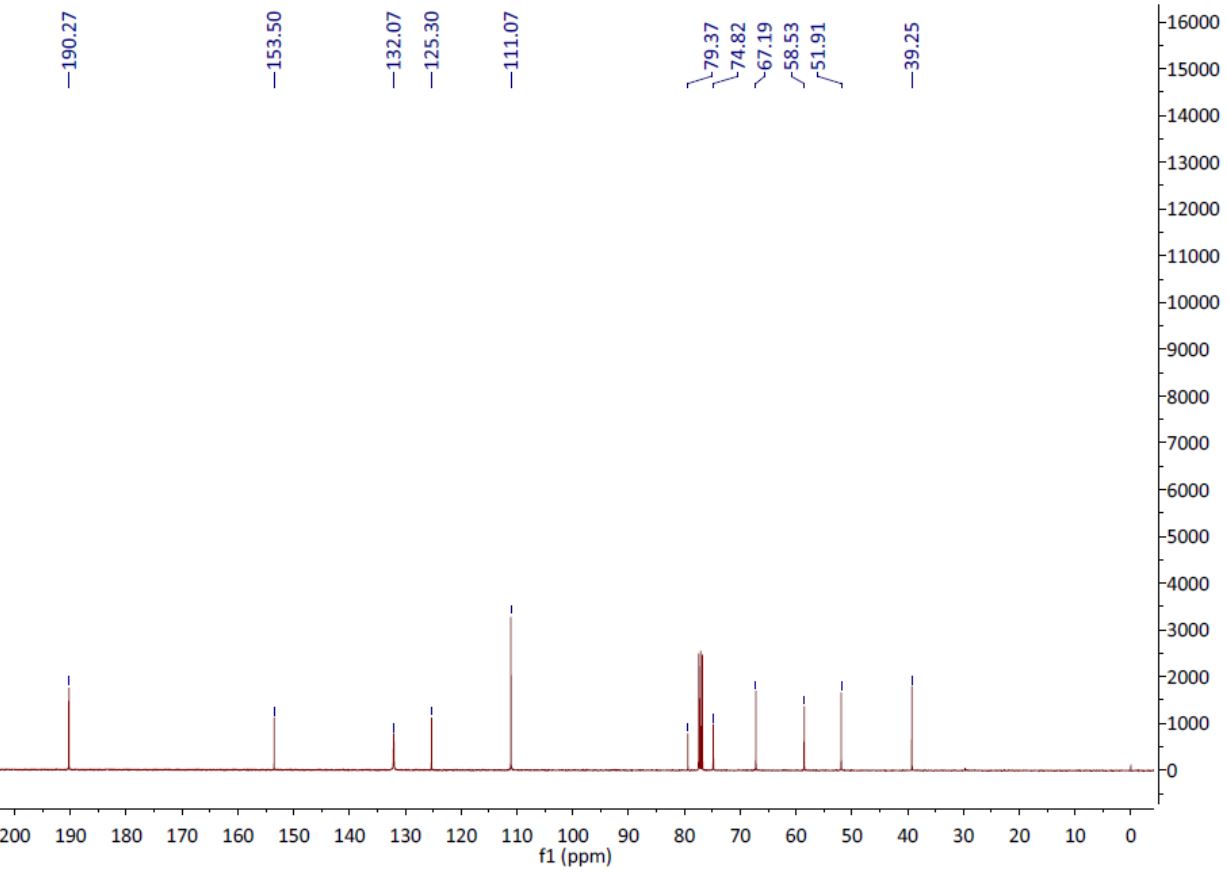
**

^13^C NMR of **c**.

**
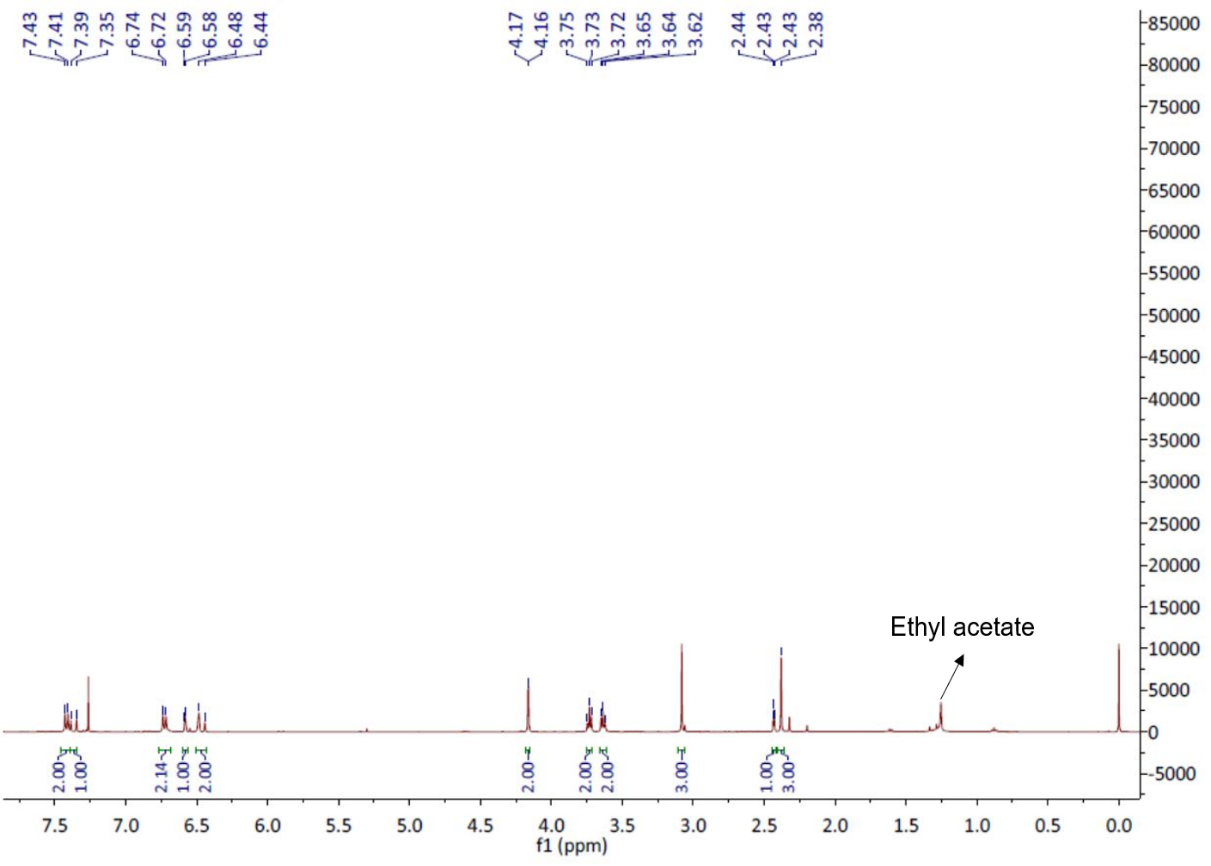
**

^1^H NMR of **e**.


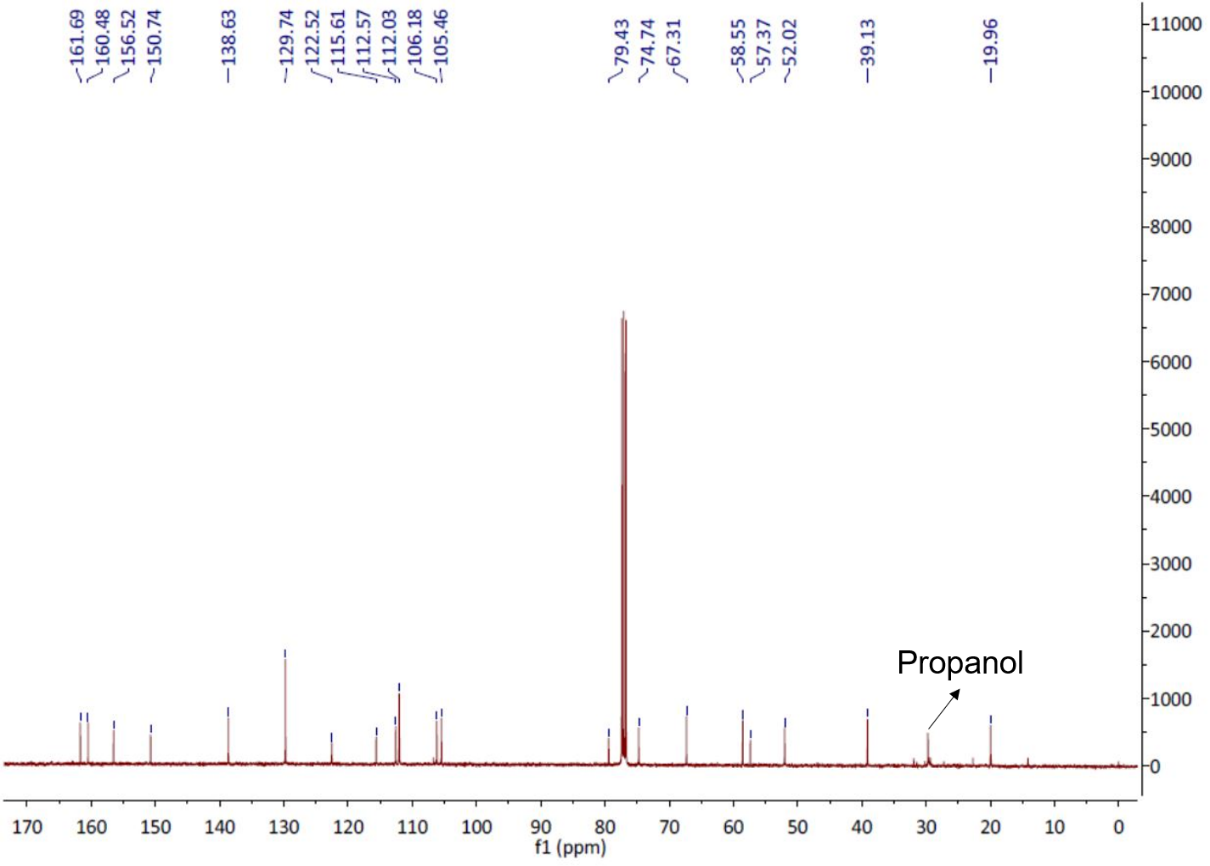


^13^C NMR of **e**.

**
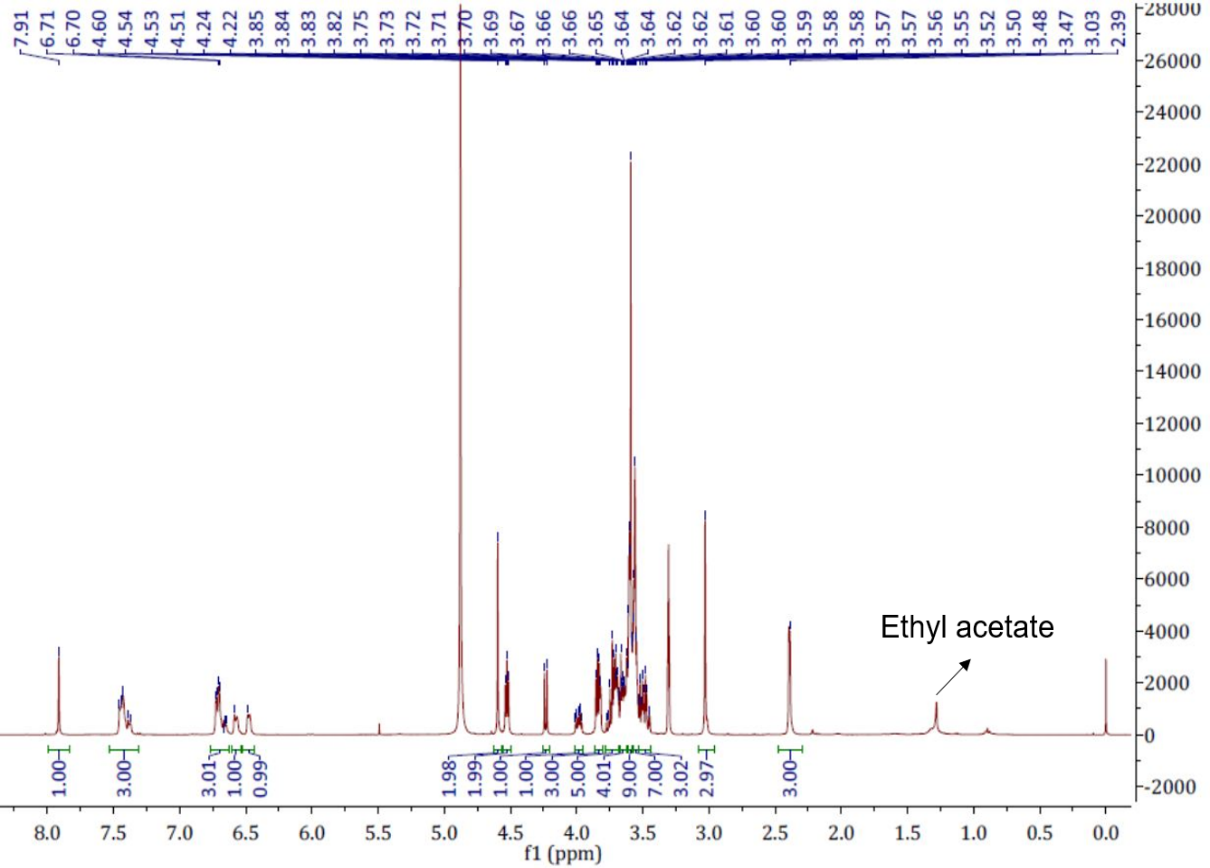
**

^1^H NMR of **g**.


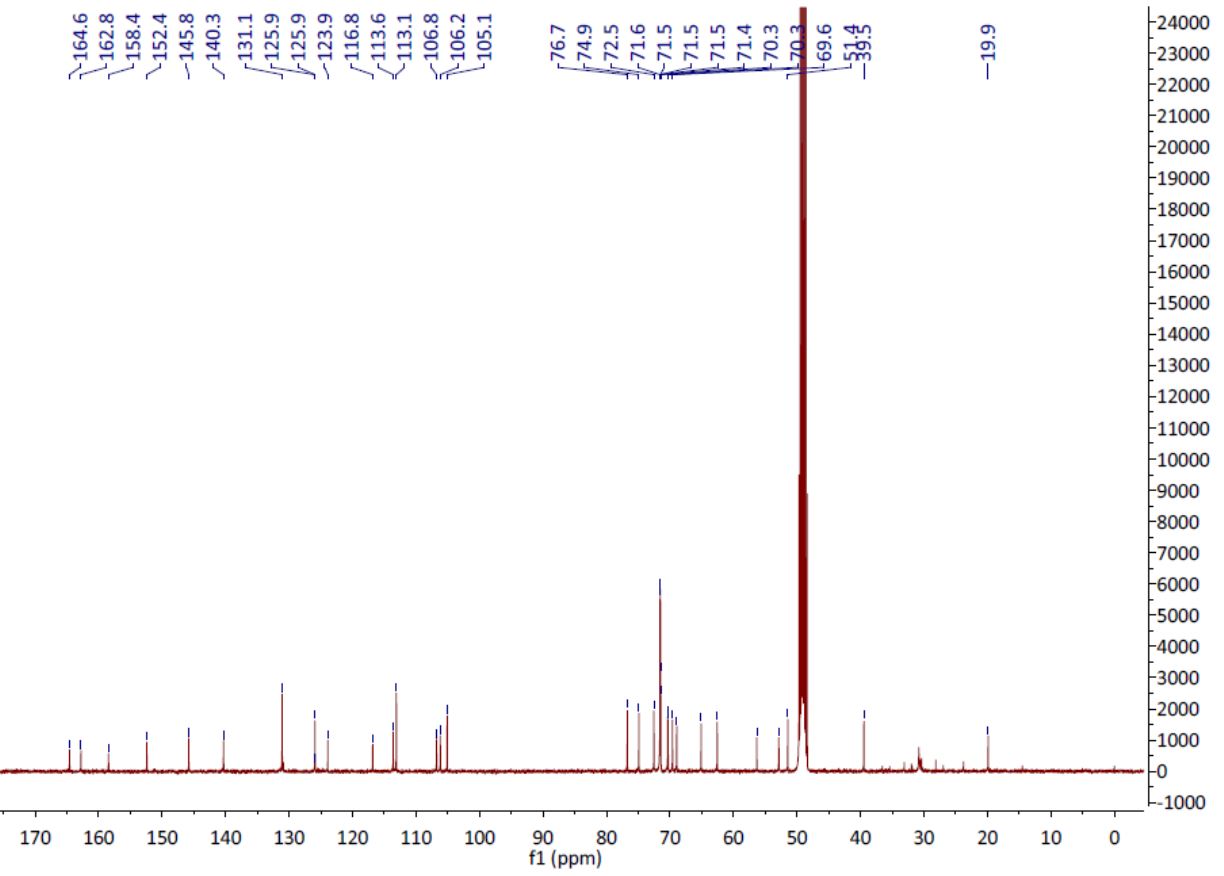


^13^C NMR of **g**.

**S3. Additional references**

Hu, X.L., Zang, Y., Li, J., Chen, G.R., James, T.D., He, X.P., et al. (2016). Targeted multimodal theranostics via biorecognition controlled aggregation of metallic nanoparticle composites. *Chemical Science* 7(7)**,** 4004-4008. doi: 10.1039/c6sc01463a.

Yan, Q., Fang, Y.C., Jia, Y.X., and Duan, X.H. (2017). Chemoselective hydrogen peroxide oxidation of primary alcohols to aldehydes by a water-soluble and reusable iron(III) catalyst in pure water at room temperature. *New Journal Of Chemistry* 41(6)**,** 2372-2377. doi: 10.1039/c6nj03793c.

Zhao, Z.L., Fan, H.H., Zhou, G.F., Bai, H.R., Liang, H., Wang, R.W., et al. (2014). Activatable Fluorescence/MRI Bimodal Platform for Tumor Cell Imaging via MnO2 Nanosheet-Aptamer Nanoprobe. *Journal Of the American Chemical Society* 136(32)**,** 11220-11223. doi: 10.1021/ja5029364.
